# The variation degree of coagulation function is not responsible for extra risk of hemorrhage in gestational diabetes mellitus

**DOI:** 10.1002/jcla.23129

**Published:** 2019-11-27

**Authors:** Chang Dong, Xiaoqiong Gu, Fei Chen, Yanlan Long, Dan Zhu, Xia Yang, Xiu Qiu, Guoquan Gao, WeiWei Qi

**Affiliations:** ^1^ Program of Molecular Medicine, Affiliated Guangzhou Women and Children's Medical Center Zhongshan School of Medicine Sun Yat‐sen University Guangzhou China; ^2^ Department of Biochemistry Zhongshan School of Medicine Sun Yat‐sen University Guangzhou China; ^3^ Guangdong Engineering & Technology Research Center for Gene Manipulation and Biomacromolecular Products (Sun Yat‐sen University) Guangzhou China; ^4^ Division of Birth Cohort Study Guangzhou Women and Children's Medical Center Guangzhou Medical University Guangzhou China; ^5^ Guangdong Province Key Laboratory of Brain Function and Disease Zhongshan School of Medicine Sun Yat‐sen University Guangzhou China

**Keywords:** coagulation function, diabetic complications, gestational diabetes mellitus, hemorrhage

## Abstract

**Background:**

Gestational diabetes mellitus (GDM) is characterized as glucose intolerance of any degree that begins or first diagnosed during pregnancy. It possesses a higher risk of haemorrhage, which may be caused by the coagulation dysfunction. However, there has been no study focus on how coagulation state changes in the progress of GDM pregnancy. Our study is aimed to assess the association of coagulation function and haemorrhage in GDM.

**Methods:**

A total of 662 subjects (273 from a population‐based study and 389 from a prospective cohort study) were selected to measure mean platelet volume (MPV), platelet distribution width (PDW), platelet (PLT), thrombocytocrit (PCT), prothrombin time (PT), activated partial thromboplastin time (APTT), thrombin time (TT), and fibrinogen (FIB). All pregnant individuals were divided into normal glucose tolerance (NGT) controls and GDM patients diagnosed between the 24th and 28th weeks of gestation.

**Results:**

Compared with NGT controls, GDM females showed shortened PT, shortened APTT, and increased blood FIB levels, while the platelet parameters MPV, PDW, PLT, and PCT remained unchanged in mid‐pregnancy. By late pregnancy, the platelet parameters MPV, PDW, and PCT were increased in the GDM group compared with the NGT group, while PT and APTT were unchanged.

**Conclusions:**

The GDM group was hypercoagulable compared with the NGT group rather than hypocoagulable as predicted, but still within the normal range. Therefore, our findings demonstrate that the variation degree of coagulation function is not responsible for extra risk of hemorrhage in GDM, and prevention of hemorrhage should focus on other causes.

**Abbreviations**
APTT: activated partial thromboplastin time
FIB: fibrinogen
GDM: gestational diabetes mellitus
MPV: mean platelet volume
NGT: normal glucose tolerance
OGTT: oral glucose tolerance test
PCT: thrombocytocrit
PDW: platelet distribution width
PLT: platelet
PT: prothrombin time
TT: thrombin time

## 1 INTRODUCTION

Gestational diabetes mellitus (GDM) is characterized by glucose intolerance of any degree that begins or is first diagnosed during pregnancy, not including diabetes occurring before pregnancy.^1^ The morbidity of GDM is approximately 11%‐12% in China, and its incidence has increased by an estimated 30% in the past decade.^2^ Notably, GDM is a common metabolic disease with variable prevalence rates between 0.6% and 15% worldwide, and it aggravates the risk of severe perinatal complications, such as massive hemorrhage, preeclampsia, shoulder dystocia, and macrosomia, for both mothers and offspring.^3^

Blood coagulation and platelet‐mediated primary hemostasis have evolved as significant defense mechanisms to stop bleeding.^4^ The anticoagulation system ensures careful control of coagulation to maintain the natural balance of physiological conditions.^5^ In the cascade of hemostasis, activated platelets can transform prothrombin into thrombin, which continues to convert fibrinogen into fibrin.^6^ There are several major changes in the hemostatic system in normal pregnancy, including increases in factors I, II, VII, VIII, IX, and XII and plasma fibrinogen and decreases in the amount of protein S, the activity of activated protein C, and the occurrence of fibrinolysis.^7^ Moreover, platelets are activated in normal pregnancy, especially before delivery.^8^ All together, these changes cause physiological hypercoagulation in normal pregnancy, which is considered to be a maternal adaption to delivery.^9^ A systematic study showed that hemorrhage was still the major cause of maternal death worldwide, with a proportion of 27.1% and responsible for 35.8% of maternal deaths in Eastern Asia, but only 16.3% in developed countries.^10^ The incidence of maternal hemorrhage is higher in developing countries than in developed countries, which means hemorrhage remains a challenging social medical problem in developing regions.^10^ Obstetric hemorrhage can be divided into antepartum, intrapartum, and postpartum hemorrhage, and the common causes of hemorrhage include maternal coagulation disorders, uterine atony, genital tract trauma, uterine rupture, or retained placental tissue.^11^

Although coagulation function has been well studied in normal pregnancy,^7^ the coagulation state of GDM patients is not well explained. It has been reported that GDM females have 3‐5 times higher risk of hemorrhage than normal glucose tolerance (NGT) females.^12^ Therefore, it is reasonable to predict that GDM possesses the potential for hypocoagulability, which may contribute to the occurrence of hemorrhage.

Whether the extra risk of hemorrhage in GDM is related to its coagulation function still needs further study. Moreover, there has been no study focusing on how the coagulation state changes in the progress of GDM pregnancy. Therefore, our study aimed to determine whether NGT females and GDM females differed in blood coagulation state and how blood coagulation state contributed to the occurrence of hemorrhage.

## 2 MATERIALS AND METHODS

### 2.1 Population‐based study

The population‐based study was performed by Guangzhou Women and Children's Medical Center (GWCMC). Four time points were set up: nonpregnancy (32 cases), mid‐pregnancy (gestational weeks 22‐28) (76 cases), late pregnancy (gestational weeks 35‐40) (86 cases), and postpartum (6‐8 weeks after delivery) (79 cases). Baseline information and laboratory findings for all subjects were collected. The nonpregnancy subjects were selected as healthy, childbearing age women. GDM was diagnosed by 75 g oral glucose tolerance test (OGTT). Subjects with other gestational comorbidities, such as gestational hypertension or intrahepatic cholestasis of pregnancy (ICP), were ruled out of the study. Subjects diagnosed as GDM did not receive medication or insulin treatment. According to the results of GDM diagnosis, subjects in mid‐pregnancy and late pregnancy were divided into GDM group and NGT group, and postpartum subjects were divided into NGT group and ex‐GDM group.

All individuals who participated in the study provided informed consent.

### 2.2 Prospective cohort study

A total of 389 subjects, including 192 NGT females and 197 GDM females, were selected from a prospective cohort study established by GWCMC. This cohort study was a part of the Born in Guangzhou Cohort Study (BIGCS), which aimed to reveal the influences of exposures during pregnancy on fetuses and offspring. The details of BIGCS have been described previously.^13^ In short, females over 18 and below 40 years old whose first antenatal care was performed at GWCMC were recruited into the cohort study. Questionnaires were given at first antenatal care to gather baseline information, including age, cigarette smoking, family history, personal medical history, and history of macrosomia. Clinical information was collected, including body mass index (BMI), blood pressure, fasting plasma glucose, and insulin concentration. Blood samples were taken at first antenatal care (12 to 27 gestational weeks) and third trimester (28‐40 gestational weeks); extra plasma samples were collected and stored for future use. OGTT was conducted between 24 and 28 gestational weeks and postpartum. Women with pre‐existing diabetes, metabolic disorder, cardiovascular diseases, chronic inflammatory disease, immunological diseases, or receiving any ongoing treatment were excluded from the study.

All individuals who participated in the study provided informed consent.

### 2.3 GDM Diagnosis

Gestational diabetes mellitus diagnoses strictly followed International Association of the Diabetes in Pregnancy Study Groups (IADPSG) diagnostic criteria: 75 g OGTT performed between 24 and 28 gestational weeks, with plasma glucose measured at 0 hour (5.1 mmol/L [92 mg/dL]),1 hour, 10.0 mmol/L [180 mg/dL], and 2 hours, (8.5 mmol/L [153 mg/dL]). GDM was diagnosed when one or more values were equal to or exceeded the reference range.

### 2.4 Laboratory analysis

Laboratory data, including mean platelet volume (MPV), platelet distribution width (PDW), platelet (PLT), thrombocytocrit (PCT), prothrombin time (PT), activated partial thromboplastin time (APTT), thrombin time (TT), and fibrinogen (FIB), were collected from the GWCMC database. Patients were identified by exclusive patient ID.

Platelet parameters (MPV, PDW, PLT, and PCT) analysis: Subjects were required fasting overnight. Venous blood was collected at least 0.5 mL into vacuum blood collection tube with 15% K3 EDTA. Collected blood was analyzed by Automatic Blood Cell Analyzer (Abbott Laboratories).

Coagulation parameters (PT, APTT, TT, and FIB) analysis: Subjects were required fasting overnight. Venous blood was collected at least 1.8 mL into vacuum blood collection tube with sodium citrate (0.109 mmol/L). Collected blood was analyzed by Automatic Coagulation Analyzer (STAGO STACompact).

The normal ranges of the above parameters were determined according to the hospital's criteria: MPV: 7.6‐13.2 fL; PDW: 14.8‐17.2 (10 gsp); PLT: 100‐320 (10^9^/L); PCT: 0.1%‐0.5%; PT: 11‐15 seconds; APTT: 28‐45 seconds; TT: 14‐21 seconds; and FIB: 2.0‐4.0 g/L.

### 2.5 Statistical analysis

Descriptive statistical analysis was performed using SPSS 22.0. Data distribution was determined by the Shapiro‐Wilk test. To describe continuous variables, we used the mean ± SD for normal distributions and interquartile ranges and the median for abnormal distributions. To compare the characteristics of subjects, we used *chi‐squared* test for categorical variables and Student's *t* tests and the Mann‐Whitney *U* Test for numeric variables. Inter class comparisons of continuous variables were performed by paired t tests. A *P*‐value <.05 was considered to be statistically significant in all tests.

## 3 RESULTS

Our results included two individual studies, the population‐based study and the prospective cohort study. The two studies were performed, and data were analyzed. We first analyzed data collected from the population‐based study. As shown in Figure S1, MPV and PDW, which represented platelet activity, increased with progression of the pregnancy and decreased rapidly after delivery. On the other hand, PLT manifested an opposite trend, decreasing with progression of the pregnancy and quickly being restored after delivery. These results suggested that platelets were physiologically activated in normal pregnancy.

It is known that GDM patients possess a higher risk of hemorrhage.^12^ We wondered whether platelets and coagulation function contributed to the occurrence of hemorrhage in GDM. To this end, we compared clinical platelet and coagulation parameters in both groups in mid‐pregnancy and in late pregnancy from the population‐based study. The results showed PT (NGT 12.4 seconds vs GDM 12.2 seconds, *P* = .0023) and APTT (NGT 34.7 seconds vs GDM 32.1 seconds, *P* < .0001) were elevated in mid‐pregnancy (Table 1) but remained within the normal pregnant range (PT: 11‐15 seconds; APTT: 28‐45 seconds). Furthermore, in late pregnancy, PDW (NGT 14.0 10 gsp vs GDM 15.2 10 gsp, *P* = .046) was slightly elevated compared with NGT, but there was no sign of change in coagulation parameters (Table 1). Moreover, the platelet parameters showed no change according to the postpartum data (Table S1). The results above gave us a primary impression as to how coagulation function changed in the progress of GDM.

**Table 1:** Platelet and Coagulation parameters in population‐based study

	Mid‐pregnancy			Late pregnancy		
	NGT (n = 31)	GDM (n = 47)	*P*‐value	NGT (n = 45)	GDM (n = 39)	*P*‐value
APTT (s)	34.7 ± 2.92	32.1 ± 2.25	**<.0001**	32.1 ± 3.55	3.5 ± 2.75	.138
PT (s)	12.4(0.625)	12.2(0.725)	**.023**	12.3 ± 0.50	12.3 ± 0.51	.879
TT (s)	16.5(1.1)	16.35(0.85)	.602	16.4(1.15)	16.5(1.3)	.689
FIB (g/L)	4.1689 ± 0.56	4.1689 ± 0.59	**.028**	4.55(0.96)	4.78(0.965)	.239
MPV (fL)	10.6(1.275)	10.25(1.175)	.07	11.0 ± 1.09	10.8 ± 1.61	.681
PLT (10^9^/L)	225.5(61)	238(73.5)	.431	219.5(57)	197(81.5)	.085
PDW (10 gsp)	12.4 ± 1.85	13.3 ± 2.18	.106	14.0 ± 2.26	15.2 ± 2.54	**.046**
PCT (%)	0.24±0.043	0.24±0.067	.932	0.24(0.08)	0.21(0.08)	**.028**

Note

Significant *P*‐values are printed in bold font.

Abbreviations: APTT, activated partial thromboplastin time; FIB, fibrinogen; GDM, gestational diabetes mellitus; MPV, mean platelet volume; NGT, normal glucose tolerance; PCT, thrombocytocrit; PDW, platelet distribution width; PLT, platelet; PT, prothrombin time; TT, thrombin time.

Therefore, to further determine GDM's coagulation state, we expanded our observation by adding 192 NGT females and 197 GDM females from a prospective cohort study. The characteristics of the NGT group and the GDM group are shown in Table 2. The two groups showed differences in pregnancy in BMI (*P* = .046) and gestational age (*P* < .0001) but no differences in family history of diabetes (*P* = .097), smoking (*P* = .078), and parity (*P* = .417).

**Table 2:** Baseline Characteristics of cohort study women

	NGT (n = 192)	GDM (n = 197)	*P*‐value
pregnancy BMI (kg/m^2^)	20.4 ± 3.1	21.0 ± 2.8	**.046**
Age (years)	28.7 ± 3.12	30.45 ± 3.35	**<.0001**
Family History of Diabetes	192/21	197/33	.097
Smoking	192/3	198/0	.078
**Parity**			
Nulliparous (%)	88.5	85.8	.417
One or greater (%)	11.5	14.2	

Note

Significant *P*‐values are printed in bold font.

Abbreviations: BMI, body mass index; GDM, gestational diabetes mellitus; NGT, normal glucose tolerance.

The clinical characteristics and laboratory findings of mid‐pregnancy are shown in Table S2. There were significant differences in OGTT gestational weeks (*P* = .037), gestational weight gained (*P* < .0001), systolic pressure (*P* = .034), fasting plasma insulin (FPI) (*P* = .012), and homeostasis model assessment (HOMA‐IR) (*P* = .001), while there were no differences in diastolic pressure (*P* = .188) or fasting plasma glucose (FPG) (*P* = .758) in mid‐pregnancy. In late pregnancy, GDM appeared to have no influence on gestational week at delivery (*P* = .873), delivery mode (*P* = .88), fetal gender (*P* = .958), birth weight (*P* = .376), birth length (*P* = .437), or Apgar scores (1 minute, *P* = .494; 5 minutes *P* = .264) (Table S3).

Corresponding to the population‐based study's results, as shown in Table 3, in the cohort study PT (NGT 12.3 seconds vs GDM 12.1 seconds, *P* = .007) and APTT (NGT 33.8 seconds vs GDM 32.9 seconds, *P* < .0001) were both shorter than those of the NGT group in mid‐pregnancy, but no values exceeded the normal pregnant range (PT: 11‐15 seconds; APTT: 28‐45 seconds). At the same time, there were no differences in platelet parameters MPV (*P* = .142), PLT (*P* = .182), and PDW (*P* = .367) between the NGT and GDM groups (Table 3). In late pregnancy, both coagulation and platelet parameters had no significant differences, except MPV slightly increased (NGT 9.3 fL vs GDM 9.8 fL, *P* = .013) but still did not exceed the normal pregnant range (MPV: 7.6‐13.2 fL) (Table 3). These results indicated that although the differences in platelets and coagulation parameters between the two groups were statistically significant, the absolute values were within the normal range, which represented little clinical value.

**Table 3:** Platelet and Coagulation parameters of prospective cohort study

	Mid‐pregnancy			Late pregnancy		
	NGT (n = 192)	GDM (n = 197)	*p*‐value	NGT (n = 192)	GDM (n = 197)	*P*‐value
APTT (s)	33.8 ± 2.46	32.9 ± 2.37	**<.0001**	32.8 ± 2.60	32.4 ± 2.29	.26
PT (s)	12.3 ± 0.57	12.1 ± 0.57	**.007**	12.2 ± 0.62	12.2 ± 0.55	.612
TT (s)	15.9 ± 0.85	16.0 ± 0.92	.238	16.3 ± 1.10	16.5 ± 0.98	.148
FIB (g/L)	4.3 ± 0.64	4.6 ± 0.70	**.004**	4.8 ± 0.68	5.0 ± 0.70	.143
MPV (fL)	9.3 ± 1.46	9.5 ± 1.30	.142	9.3 ± 1.53	9.8 ± 1.46	**.013**
PLT (10^9^/L)	237.0 ± 52.59	245.5 ± 51.24	.182	231.0 ± 54.48	221.4 ± 53.06	.108
PDW (10 gsp)	13.9 ± 2.53	13.6 ± 2.52	.367	14.3 ± 2.75	13.9 ± 2.59	.198
PCT (%)	0.2193 ± 0.05	0.2333 ± 0.05	**.03**	0.2131 ± 0.05	0.2135 ± 0.05	.946

Note

Significant *P*‐values are printed in bold font.

Abbreviations: APTT, activated partial thromboplastin time; FIB, fibrinogen; GDM, gestational diabetes mellitus; MPV, mean platelet volume; NGT, normal glucose tolerance; PCT, thrombocytocrit; PDW, platelet distribution width; PLT, platelet; PT, prothrombin time; TT, thrombin time.

## 4 DISCUSSION

The current study aimed to clarify the association of coagulation function with GDM hemorrhage. For this purpose, we analyzed how coagulation and platelet parameters change in the progress of GDM and NGT individually and then compared the differences between two groups. As the results showed, PT and APTT were decreased and fibrinogen level was increased in mid‐pregnancy in GDM, and MPV and PDW increased in late pregnancy. Our study provided clues that the GDM group was hypercoagulable compared with the NGT group rather than hypocoagulable as predicted, but still within the normal range. Therefore, our findings demonstrate that the extra risk of hemorrhage in GDM is not due to coagulation function change, and prevention should focus on other causes.

Our study was the first to observe how platelets and coagulation parameters change in the progress of GDM pregnancy. MPV and PDW describe platelet volume and its variation; they together represent the activation degree of platelets.^14^, ^15^ PLT represents platelets count, and PCT is the product of MPV and PLT. PT and APTT reflect the extrinsic and intrinsic pathways of coagulation function, respectively. TT represents fibrinolytic system function, and FIB can reflect the state of coagulation function.^16^ Previous studies were either considered to be relatively small or failed to trace with the progression of pregnancy. In accordance with former studies,^17^ our study confirmed that platelet activity was enhanced with the progression of pregnancy and returned to normal level after delivery, while platelet counts changed conversely (Figure S1). The results showed that platelets were activated, and counts were decreased in late pregnancy. In normal pregnancy, the change is a physiological state to protect the body from hemorrhage during or after delivery.^8^ Contrary to our prediction, our results showed that despite GDM patients possessing a higher risk of hemorrhage, the GDM group tended to be hypercoagulable instead of showing compromised coagulation function. Corresponding to our results, some researchers demonstrated that the significantly increasing fibrinogen level was an indicator of GDM patients being more hypercoagulable than NGT patients.^18^, ^19^ Abdel^18^ analyzed 150 GDM females and 100 normal pregnant females in their third trimesters and found that the fibrinogen level was increased in the GDM group while APTT and PT were unchanged. Another study, launched by Suheyla,^19^ included 16 NGT females and 19 GDM females in the 20th to 24th gestational weeks and found that APTT and PT were unchanged, and the fibrinogen level was higher in the GDM group. However, neither of these studies included how GDM patients’ coagulation state changed during pregnancy progression. Other studies, such as Gumus et al,^20^ focused on the fibrinolysis system and revealed similar conclusions by showing increasing thrombin activatable fibrinolysis inhibitor (TAFI). Our results showed that the coagulation parameters APTT and PT were lowered while the fibrinogen level was increased in GDM. Nevertheless, the phenomenon only took place in mid‐pregnancy, and in late pregnancy, both PT and APTT showed no differences between the two groups. To our knowledge, in mid‐pregnancy, hypercoagulation in GDM females could be mainly caused by hyperglycemia. Nonetheless, body's adaption to delivery eliminated the differences when it came to late pregnancy. As reported by former studies, GDM could increase the chance that fetuses develop macrosomia; moreover, laboring with macrosomia could result in uterine atony and genital tract trauma.^21^, ^22^ Therefore, we believe that the extra risk of hemorrhage in GDM is mainly attributed to delivering babies with macrosomia, but not to altered coagulation function. Currently, the prevention of GDM hemorrhage in clinical practice mainly includes monitoring for macrosomia and termination of pregnancy at a proper time.^22^

Gestational diabetes mellitus is also regarded as a special condition of diabetes because its clinical manifestation is similar to type 2 diabetes mellitus (T2DM), causing hyperglycemia, insulin resistance, and impaired insulin secretion.^3^ Mounting studies reported that T2DM patients had abnormal platelet activation^23^, ^24^ and their coagulation systems were in a hypercoagulable state,^25^ which could be the pathogenesis of thrombosis, atherosclerosis, and diabetic vascular complications.^26^, ^27^ As our results show in Tables 1 and 3, platelets tended to be activated in late pregnancy in GDM (both MPV and PDW can indicate the activation of platelets). Platelets are physiologically activated (Figure S1), which could help explain hypercoagulation in late pregnancy. Furthermore, GDM as the special condition of diabetes, whose platelets were abnormally activated, indicate postpartum complications and long‐term diabetes occurrence.^28^, ^29^ For example, Ozlem^30^ analyzed platelet parameters from 30 NGT and 38 GDM females and showed that MPV and PDW were elevated in pregnancy. However, in 2017, Suheyla^31^ reported an inverse result, demonstrating that PLT was elevated and MPV was decreased in late pregnancy in GDM. In accordance with previous studies, we also believe that platelets are abnormally activated in GDM and that the degree of platelet activation might indicate postpartum or long‐term complications, such as inflammation and T2DM.

Our study provided evidence that GDM patients were hypercoagulable compared with NGT patients rather than hypocoagulable, as predicted. However, there were no clinical platelet or coagulation parameters exceed the normal pregnant range. These results suggested that the variation degree of coagulation function is not responsible for extra risk of hemorrhage in GDM, and prevention of hemorrhage should focus on other causes.

## AUTHOR CONTRIBUTION

W. Qi, G. Gao, X Qiu, and X Yang were involved in the concept and design of the study. C. Dong, X. Gu, Y. Long, and D. Zhu were responsible for conducting the experiments. C. Dong, X. Gu, and X Qiu drafted the article, and W. Qi revised the article. C. Dong and W. Qi were responsible for data analysis. All authors contributed to the interpretation of data and provided revisions to the article. W. Qi will act as guarantor for the study.

## ETHICAL APPROVAL

The study was approved by Guangzhou Women and Children's Medical Center's Medical Ethics Committee (no. 2012 [015]; date: 06/06/2013 reissued). All subjects have signed informed consent forms. The information and samples of each participant are stored in the hospital's independent database.

## INFORMED CONSENT

Informed consent was obtained from all individual participants included in the study.

## Data Availability

The patients’ characteristic, medical information, and laboratory outcomes used to support the findings of this study were supplied by Guangzhou Women and Children's Medical Center under license and so cannot be made freely available. Requests for access to these data should be made to Guangzhou Women and Children's Medical Center, 9 Jinsui Road, Guangzhou, China, 510623, +86(020)‐81886332.
