# Supplementary material for: The variation degree of coagulation function is not responsible for extra risk of hemorrhage in gestational diabetes mellitus
Source: J Clin Lab Anal. 2019 Nov 27;34(4):e23129. doi: 10.1002/jcla.23129 (PMC7171326; doi:10.1002/jcla.23129)

**Supplemental Table 1. Platelet and Coagulation parameters of postpartum in population-based study**

|  | NGT (n=29) | GDM (n=50) | *p*-value |
| --- | --- | --- | --- |
| Age (years) | 29.3±2.79 | 30.1±3.77 | 0.306 |
| PCT (%) | 0.28(0.04) | 0.27(0.0975) | 0.635 |
| PDW (10 gsp) | 13.3±2.12 | 12.8±1.68 | 0.324 |
| PLT (10^9/L) | 257.6±50.27 | 258.9±71.36 | 0.932 |
| MPV (fL) | 10.8±0.98 | 10.5±0.86 | 0.201 |

NGT: normal glucose tolerance, GDM: gestational diabetes mellitus, MPV: mean platelet volume, PDW: platelet distribution width, PLT: platelet, PCT: thrombocytocrit.

**Supplemental Table 2. Clinical characteristics and laboratory findings of prospective cohort study in mid-pregnancy**

|  | NGT (n=192) | GDM (n=197) | *p*-value |
| --- | --- | --- | --- |
| OGTT Gestational weeks | 24.7±1.35 | 24.98±1.31 | **0.037** |
| Gestational Weight Gain up to OGTT (kg) | 14.9±4.79 | 13.1±4.10 | **＜0.0001** |
| Systolic Pressure (mmHg) | 105.4±9.93 | 107.6±9.70 | **0.034** |
| Diastolic Pressure (mmHg) | 64.7±7.87 | 65.7±7.12 | 0.188 |
| OGTT 0h (mmol/L) | 4.3±0.36 | 4.7±0.54 | **＜0.0001** |
| OGTT 1h (mmol/L) | 7.3±1.59 | 10.10±1.47 | **＜0.0001** |
| OGTT 2h (mmol/L) | 6.4±1.07 | 8.6±1.39 | **＜0.0001** |
| FPG (mmol/L) | 4.4±0.46 | 4.4±0.47 | 0.758 |
| FPI (uIU/L) | 10.2±7.06 | 12.0±6.89 | **0.012** |
| HOMA_IR | 2.0±1.36 | 4.0±6.82 | **0.001** |

NGT: normal glucose tolerance; GDM: gestational diabetes mellitus; OGTT: oral glucose tolerance test; FPG: fasting plasma glucose; FPI: fasting plasma insulin; HOMA-IR: Homeostasis model assessment, calculation HOMA-IR=FPG (mmol/L) * FPI (μIU/mL)/ 22.5. Significant *p*-values are printed in bold font.

**Supplemental Table 3.** **Characteristics and pregnancy outcomes of prospective cohort study women in late-pregnancy**

|  | NGT (n=192) | GDM (n=197) | *p*-value |
| --- | --- | --- | --- |
| Gestational week at delivery | 38.8±1.20 | 38.7±1.19 | 0.873 |
| Weight gain in pregnancy (kg) | 14.9±4.79 | 13.1±4.10 | **＜0.0001** |
| **Delivery Mode** |  | | |
| Vaginal Delivery (%) | 68.2 | 67.5 | 0.88 |
| Cesarean Delivery (%) | 31.8 | 32.5 |  |
| **Fetal Gender** |  | | |
| Male (%) | 49.5 | 49.7 | 0.958 |
| Female (%) | 50.5 | 50.3 |  |
| Birth Weight (g) | 3193.2±380.5 | 3228.4±402.1 | 0.376 |
| Birth Length (cm) | 49.4±1.6 | 49.5±1.6 | 0.437 |
| Apgar 1min | 9.0±0.36 | 9.1±0.34 | 0.494 |
| Apgar 5 min | 9.10±0.35 | 9.14±0.35 | 0.264 |

NGT: normal glucose tolerance, GDM: gestational diabetes mellitus. Significant p-values are printed in bold font.

**Supplemental Figure 1. Plasma platelet parameters are changed in normal pregnancy.**


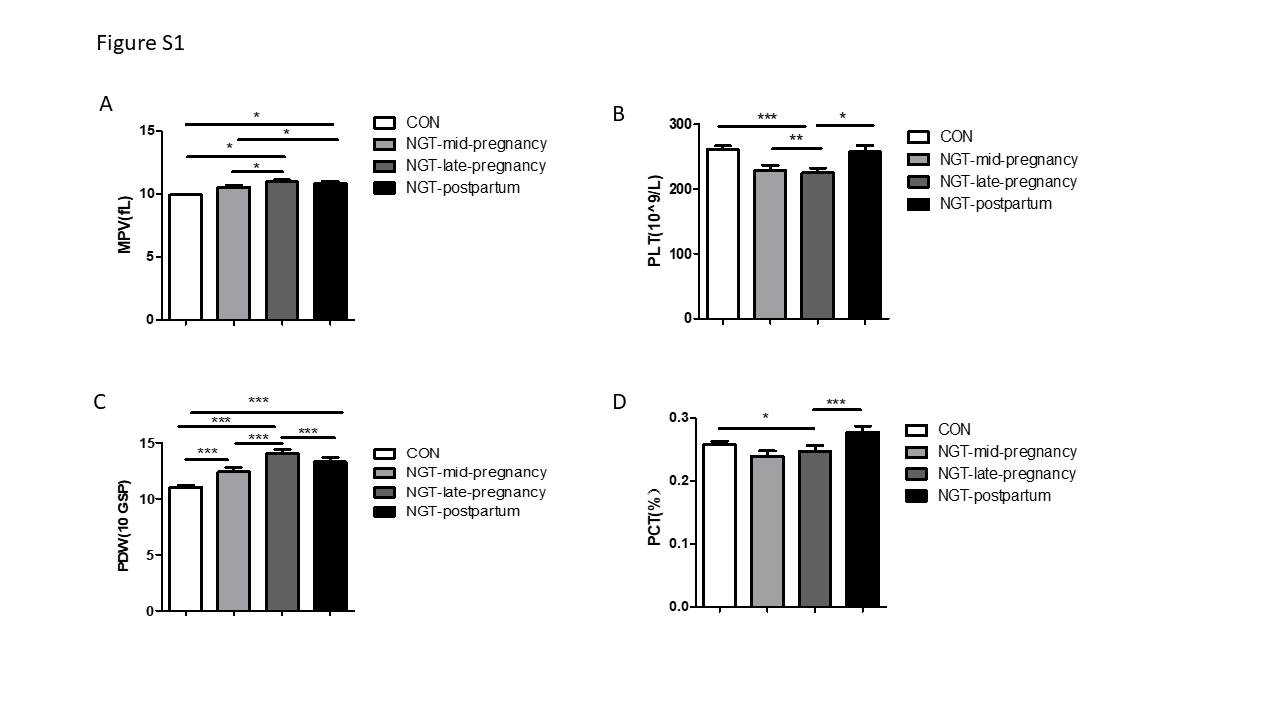

Supplement: Supplementary file 1 [file JCLA-34-e23129-s001.docx]
